# Colocynth Extracts Prevent Epithelial to Mesenchymal Transition and Stemness of Breast Cancer Cells

**DOI:** 10.3389/fphar.2017.00593

**Published:** 2017-09-05

**Authors:** Kaushik Chowdhury, Ankit Sharma, Suresh Kumar, Gyanesh K. Gunjan, Alo Nag, Chandi C. Mandal

**Affiliations:** ^1^Department of Biochemistry, School of Life Sciences, Central University of Rajasthan Ajmer, India; ^2^Department of Biochemistry, University of Delhi New Delhi, India

**Keywords:** cancer, colocynth, cell death, apoptosis, cell viability, epithelial to mesenchymal transition, metastasis

## Abstract

Modern treatment strategies provide better overall survival in cancer patients, primarily by controlling tumor growth. However, off-target and systemic toxicity, tumor recurrence, and resistance to therapy are still inadvertent hurdles in current treatment regimens. Similarly, metastasis is another deadly threat to patients suffering from cancer. This has created an urgent demand to come up with new drugs having anti-metastatic potential and minimum side effects. Thus, this study was aimed at exploring the anti-proliferative and anti-metastatic potential of colocynth medicinal plant. Results from MTT assay, morphological visualization of cells and scratch assay indicated a role of ethanol and acetone extracts of fruit pulp of the colocynth plant in inhibiting cell viability, enhancing cell cytotoxicity and preventing cell migration in various cancer cell types, including breast cancer cell lines MCF-7 and MDA-MB-231, and cervical cancer cell line SiHa, subsequently having a low cytotoxic effect on mononuclear PBMC and macrophage J774A cells. Our study in metastatic MDA-MB-231 cells showed that both ethanol and acetone pulp extracts decreased transcript levels of the anti-apoptotic genes BCL2 and BCLXL, and a reverse effect was observed for the pro-apoptotic genes BAX and caspase 3. Additionally, enhanced caspase 3 activity and downregulated BCL2 protein were seen, indicating a role of these extracts in inducing apoptotic activity. Moreover, MDA-MB-231 cells treated with both these extracts demonstrated up-regulation of the epithelial gene keratin 19 and down-regulation of the mesenchymal genes, vimentin, *N*-cadherin, Zeb1 and Zeb2 compared to control, suggesting a suppressive impact of these extracts in epithelial to mesenchymal transition (EMT). In addition, these extracts inhibited colony and sphere formation with simultaneous reduction in the transcript level of the stemness associated genes, BMI-1 and CD44. It was also found that both the plant extracts exhibited synergistic potential with the chemotherapeutic drug doxorubicin to inhibit cancer viability. Furthermore, GC-MS/MS analysis revealed the presence of certain novel compounds in both the extracts that are responsible for the anti-cancer role of the extracts. Overall, the results of this report suggest, for the first time, that colocynth fruit pulp extracts may block the proliferative as well as metastatic activity of breast cancer cells.

## Introduction

Cancer constitutes a group of deadly diseases that is not only the second leading cause of death worldwide, but also largely contributes to the global health economic burden. The World Health Organization (WHO) has estimated over 14.1 million cancer cases and 8.2 million cancer-induced deaths worldwide in 2012, and this number is expected to rise catastrophically in the coming decades (Torre et al., 2015). Breast and cervical cancers are the most frequently diagnosed cancers in females nationwide, accounting for about 1.7 million new cases and 521,900 breast cancer deaths in 2008 (Torre et al., 2015). India infamously boasts of a surmounting 27 and 23% breast and cervical cancer incidence respectively^1^. Cervical cancer is the second most diagnosed cancer and third leading cause of deaths in females, accounting for about 527600 new cases and 265700 deaths (Torre et al., 2015).

Cancer is predominantly caused due to abnormalities in the genome (Balmain et al., 2003) and epigenome (Feinberg and Tycko, 2004) due to exposure to various damaging agents. This set of accumulated cells that has escaped the normal regulatory control mechanisms undergoes unchecked proliferation to form tumors. Despite several advancements in recent years, contemporary anticancer therapy suffers from several limitations owing to their associated toxicity and off-target effects. This provokes an urgent need to design novel drugs with high efficacy specific for cancer cells and less toxicity to off-target cells. Phytochemicals have shown promise in this regard as they fit the above criteria, and their usage in anticancer therapy is an emerging trend.

*Citrullus colocynth* (L.) is a valuable cucurbit plant, widely distributed in the desert areas of the world, including India, known to possess nutritional values and diverse medicinal activities, including antibacterial, antifungal, larvicidal and anti-inflammatory properties (Sawaya et al., 1986; Marzouk et al., 2010; Chawech et al., 2017). Literature documents the presence of many bioactive compounds, such as cucurbitacin, phenolic acids, flavonoids, pyridine and quinolone type alkaloids and fatty acids in fruits of these herbal plants (Hussain et al., 2013, 2014; Jeon and Lee, 2014). This plant is traditionally used to control diabetes (Shi et al., 2014). Recent clinical trial studies have witnessed a fall in fasting blood glucose and Hb1Ac, triglyceride and cholesterol in case of colocynth users (Rahbar and Nabipour, 2010; Barghamdi et al., 2016). Intriguingly, a study by Tannin-Spitz et al. (2007) documented cancer specific apoptotic activity of the isolate cucurbitacin, extracted from this plant. However, no study has yet been conducted to explore the effect of colocynth extract in cancer metastasis. Thus, this study was primarily aimed at investigating the unexplored anti-metastatic potential of this plant extract.

This study testified that ethanol and acetone fruit pulp extracts exhibited impressive inhibition of cell viability and cell migration of various cancer cell types, including breast and cervical cancer cells with considerably less effect on mononuclear cells and macrophage cells. Moreover, these pulp extracts noticeably hindered colony and sphere formation and epithelial to mesenchymal transition (EMT) of metastatic breast cancer MDA-MB-231 cells. Our GC-MS analysis also reveals some unique compounds, which may account for the anticancer activity of the extracts. The current study is the first report advocating that fruit pulp extracts containing the novel compounds may have anti-metastatic potential along with apoptotic activity.

## Materials and Methods

### Materials

Verso cDNA synthesis kit (AB1453A, Thermo Scientific), TRIzol Reagent (T9424, Sigma Aldrich), Taq Polymerase (MBT060A, Himedia), ready Mix dNTP (MBT078, Himedia), caspase-3 antibody (#9661, Cell signaling), BCL-2 antibody (SC-7382, Santa Cruz Technology), actin antibody (A02066, Sigma Aldrich), WesternSure-Premium Chemiluminescent substrate (WesternSure-Li-COR-Part No: 926-95000).

### Cell Lines

The human breast cancer MDA-MB-231 (metastatic) and MCF-7 (non-metastatic) cell lines, and cervical cancer SiHa cell line were procured from NCCS cell repository, Pune, India. J774A cell (Macrophage cell line) was obtained from Dr. Vijay Kumar Prajapati, Department of Biochemistry, Central University of Rajasthan, India. All cells were cultured in Dulbecco’s Modified Eagles Medium (DMEM), supplemented with 10% fetal bovine serum (FBS) (RM1112, Himedia) and maintained at 37°C in a humidified incubator with 5% CO_2_.

### Isolation of Human Mononuclear Cells (PBMC)

Mononuclear cells were isolated from human peripheral blood by using a simple and rapid density gradient centrifugation technique using Ficoll-Paque (Sigma-F5414-50ML) methodology established by Boyum (1968) and Boyum (1976). The isolation was done according to the manufacturer’s protocol. Cells (0.5 × 10^5^ cells) were seeded in 96 well culture plate in DMEM supplemented with 10% FBS and incubated for overnight at 5% CO_2,_ and treated with increasing concentration (mentioned in other cell lines) by ethanolic and acetone plant extracts respectively for 24 h. Blood samples from two healthy volunteers were taken and mixed before isolation of PBMC. Written consent was obtained from the participants, and they were informed about the use of blood in this study. Moreover, the work related to blood samples had been conducted by following the regulation of Institutional Ethical Committee at Central University of Rajasthan and, this study was approved by Institutional Ethical Committee.

### Plant Extracts

The plant *Citrullus colocynth* was obtained from a rural area of India [Jaisalmer (26.9157° N, 70.9083° E), Rajasthan, India]. The taxonomic name of this plant had been confirmed by Dr. Amit Kotia, Department of Botany, University of Rajasthan, India. The pulp was isolated from fruits, dried and crushed, using a mixer grinder. One gram of dried pulp powder was suspended in 10 ml of ethanol and acetone, followed by solvent extraction at 50°C for 72 h. The solvent fractions were evaporated using a rotary-evaporator at 42°C to get the crude products. The crude extracts obtained were then dissolved in ethanol and acetone and kept at -20°C for further use.

### MTT Cell Viability Assay

Cell viability was measured by MTT assay (MB186, Himedia) described earlier (Ghosh-Choudhury et al., 2010; Mehta et al., 2015). In brief, cancer cells (5000 cells/well) were seeded in 96 well tissue culture plate in DMEM supplemented with 10% FBS at an atmosphere of 5% CO_2_ at 37°C. After 24 h of seeding, cells were separately incubated with different concentrations [25, 100, and 250 μg/ml; equivalent to 1, 4, and 10 μl of solvents (ethanol/acetone)] of fruit pulp ethanol extract (PEE) and pulp acetone extract (PAE) for 24 h. Control cells were similarly incubated with equal volume of the solvents as in the experimental wells. At the end of the incubation period, 10 μl of MTT solution (5 mg/ml) was added to each well and the plate was incubated at 37°C for 1 h. The formazan crystals formed were solubilized by adding DMSO, and subsequently, absorbance was measured at a wavelength of 530 nm (Studzinski, 1995; Moongkarndi et al., 2004; Ghosh-Choudhury et al., 2010; Mehta et al., 2015). The effective absorbance was calculated for the experimental wells with respect to corresponding control wells. LC_50_ (Lethal concentration at which 50% cells are killed) at 24 h duration was calculated using MTT data set for different cancer cell lines (Zhang et al., 2007).

### Scratch Assay

Cell migration was determined by scratch assay as described earlier (Mandal et al., 2010b, 2011b). In brief, cells (2 × 10^5^ cells/plate) were seeded in 35 mm tissue culture plates and allowed to grow till they reach 95% confluency. A cell scratch-wound was generated using a micropipette tip, and washed once with PBS buffer to remove the floating cells. Cells were then separately treated with PEE and PAE (25 μg/ml) for 24 h in experimental plates, and equal volume of respective solvents was added in control plates. Cells were visualized under an inverted microscope (Carl-Zeiss), and cell migration was assessed by measuring gap sizes in multiple fields and the relative area calculated using ImageJ, and statistically analyzed (Hirsch et al., 2009).

### RT-PCR Analysis

Transcript levels of several genes were measured by semi-quantitative RT-PCR analysis as described previously (Mandal et al., 2010a; Chowdhury et al., 2017). In brief, breast cancer cells (2 × 10^5^ cells/plate) were plated in 35 mm tissue culture plates in DMEM supplemented with 10% FBS at an atmosphere of 5% CO_2_ at 37°C. After 90-100% cell confluency is reached, the cells were treated with different test factors (such as PEE and PAE (50 μg/ml)) for 24 h. Total RNA was extracted using TRIzol reagent as described earlier (Mandal et al., 2010a, 2011a, 2016a; Ghosh-Choudhury et al., 2013). First stand cDNA synthesis was performed using 1 μg of total RNAs using the Verso cDNA synthesis kit, according to manufacturer’s instruction. Semi-quantitative PCR was performed using this cDNA as template and gene specific primers in a ProFlex PCR system (Applied Biosystem by Life Technologies). The list of primers used in this study is given in Supplementary Table S1. Densitometric analysis of the PCR bands was done using ScnImage (Chowdhury et al., 2017).

### Western Blot Analysis

Methodology of western blotting has been described previously (Chowdhury et al., 2017). In brief, cells were seeded at a density of 2 × 10^5^ cells/plate in 35 mm tissue culture plates. Cells were treated with different test factors and harvested after 24 h of treatment. Protein extraction was carried out in RIPA buffer and subsequently, cell lysates were centrifuged at 12,000 × *g* at 4°C for 20 min. The protein concentration was measured by Bradford reagent (BIORAD), according to manufacturer’s instruction. Equal amounts of whole cell lysates were resolved by 12% SDS-PAGE, and transferred to PVDF membrane. This PVDF membrane was incubated with primary antibody (Anti-caspase-3 antibody (#9661, Cell signaling), Anti-BCL-2 antibody (SC-7382, Santa Cruz Technology), Anti-actin antibody (A02066, Sigma Aldrich) used at 1:1000 dilution) overnight at 4°C. After washing, the PVDF membrane was further incubated with HRP-conjugated secondary antibody (used at 1:10000 dilution) for 1 h at room temperature. The washed membrane was further incubated with chemiluminescent substrate and subsequently, the blot was scanned by C-DIGIT Blot Scanner (Li-COR-Model: 3600).

### Colony Formation Assay

Cells were seeded at a density of 500 cells/well in a 12 well tissue culture plate in DMEM with 10% FBS at 37°C incubator with 5% CO_2_ for 24 h. Cells were then treated with PEE and PAE extracts (25 μg/ml), and incubated under same conditions for 5 days. Next, colonies were fixed with methanol and were stained with crystal violet solution (Hirsch et al., 2009). Photographs of stained plate were taken by a camera. Photographs of colonies were also taken by a bright field inverted microscope (Carl-Zeiss).

### Soft Agar Sphere Formation Assay

For performing soft agar assay (Sebolt-Leopold et al., 1999), MDA-MB-231 cells (10,000 cells/plate) were mixed with 2X DMEM containing 0.6% (w/v) agar and layered with 2X DMEM containing 1.2% base agar medium in 35 mm culture plates. 25 μg/ml of PEE and PAE was added in 300 μl of DMEM to the top agar and cells were allowed to grow for 8 days. The top 300 μl medium was replaced with fresh medium with or without test factors at intervals of every 3 days. The morphology and size of the spheres were visualized using bright field inverted microscope (Carl-Zeiss) and photographed. Subsequently, spheres were manually counted under the microscope.

### GC-MS/MS Analysis

Solvent Extraction of *Citrullus colocynthis* fruit pulp was done by ethanol and acetone. Dried extracts were dissolved in methanol followed by GC-MS/MS analysis using Thermo GC 1300 and “TSQ 8000” Triple quadrupole GC-MS/MS SYSTEM with auto sampler Al 1310. The Gas Chromatography 1300 was used with a fused GC column TG-5MS AMINE. The column length was of 30 m with an internal diameter coated film of 0.25 μm, with the flow rate of 10 ml/min and the condition kept were as follows: PTV Temp. Program: 70°C, hold 2.00 min, 10°C/min to 270°C, hold 8 min. Carrier gas helium flow rate was 1 ml/min and split ratio of 1:50. GC was equipped with an auto sampler AI 1300 and sample volume loaded was 1 μl. Eluates were automatically passed into the mass spectrometer. Mass Spectrum analysis was conducted using TSQ-8000 with a transfer line temperature of 280°C and ion source temperature of 230°C in EI mode. Mass scan time was 4 min with feature of full Scan MS. The mass spectrum was also equipped with a computer fed NIST mass Spectra data library.

### Component Identification

Chemical constituents of the extracts were identified by matching the peaks with Computer NIST MS libraries and confirmed by comparing mass spectra of the peaks and those from literature. List of 50 compounds isolated from the extracts are listed in Supplementary Tables S2, S3. Scifinder was used to analyze the compounds and thus interpret the literature available for them.

### Statistical Analysis

Statistical analysis was done by GraphPad Prism (Ghosh-Choudhury et al., 2010; Mandal et al., 2011a,b, 2016b). A *p*-value of < 0.05 was considered to be statistically significant. All values were represented as mean ± standard error mean of three measurements.

## Results

### Effect of Colocynth Fruit Pulp Extracts on Viability of Breast and Cervical Cancer Cells

**FIGURE 1:**
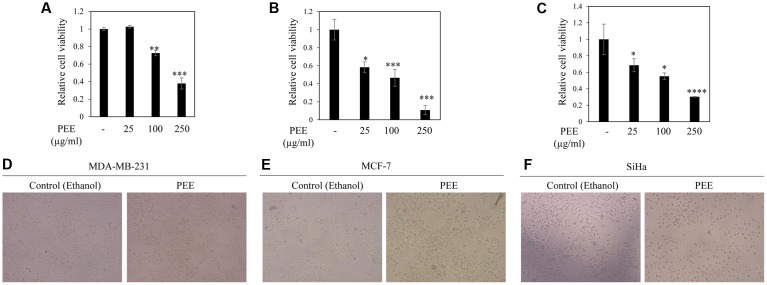
Effect of fruit pulp ethanol extract of colocynth on cell viability and cytotoxicity of breast and cervical cancer cell lines. Cell viability was measured by MTT assay after 24 h of treatment of breast cancer MDA-MB-231 **(A)** and MCF-7 **(B)**, and cervical SiHa **(C)** cell lines with pulp ethanol extract (PEE) with different concentrations (25–250 μg/ml). Values represent mean ± SE of triplicate measurements, ^∗^*p* < 0.05, ^∗∗^*p* < 0.01, and ^∗∗∗^*p* < 0.001 vs. control. **(D–F)** Morphologic visualization (cytotoxicity) was recorded after 24 hr of treatment of breast cancer MDA-MB-231 **(D)** and MCF-7 **(E)**, and cervical SiHa **(F)** cell lines with PEE (100 μg/ml). Photographs were taken at 10X magnification.

**FIGURE 2:**
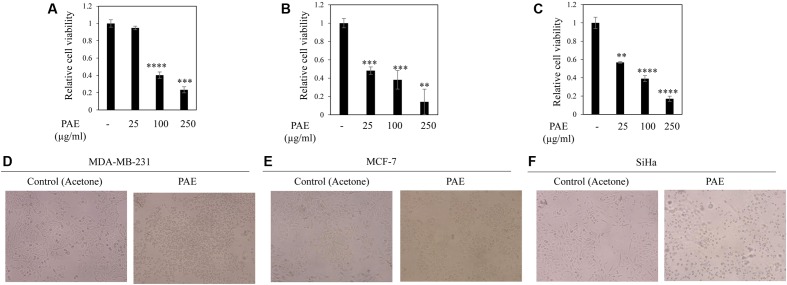
Effect of fruit pulp acetone extract of colocynth on cell viability and cytotoxicity of breast and cervical cancer cell lines. MTT assay was performed to measure cell viability by after 24 h of treatment of breast cancer MDA-MB-231 **(A)** and MCF-7 **(B)**, and cervical SiHa **(C)** cell lines with pulp acetone extract (PAE) with different concentrations (25–250 μg/ml). Values represent mean ± SE of triplicate measurements, ^∗∗^*p* < 0.01, ^∗∗∗^*p* < 0.001, and ^∗∗∗∗^*p* < 0.0001, vs. control. **(D–F)** Morphologic visualization (cytotoxicity) was recorded after 24 h of treatment of breast cancer MDA-MB-231 **(D)** and MCF-7 **(E)**, and cervical SiHa **(F)** cell lines with PAE (100 μg/ml). Photographs were taken at 10X magnification.

To test the anticancer potential of colocynth fruit pulp extracts (prepared separately by solvent ethanol and acetone), cell viability was measured by MTT assay. For this, different cancer cells, including breast cancer cell lines, MCF-7 and MDA-MB-231, and cervical cancer cell line, SiHa, were separately treated with increasing concentrations (range 25–250 μg/ml) of PEE and PAE for 24 h (**Figures 1A**–**C**, **2A–C**). MTT assay result showed noteworthy reduction in the number of viable cells in a dose dependent manner for both PEE and PAE treatment when compared to their respective solvent controls. To determine the effectiveness of the pulp extracts in these cancer cells, LC_50_ values (using MTT data) were also calculated. In case of ethanol extract, LC_50_ (μg/ml) values were 142 ± 16, 105 ± 8.3 and 157 ± 4.3 for MDA-MB-231, MCF-7 and SiHa cell lines respectively (**Table 1**). For acetone extract, these values were 140 ± 0.8, 94 ± 6.4 and 121 ± 4.6 respectively. These findings indicated relatively higher efficacy of PAE in cancer cell lines compared to PEE. Moreover, MCF-7 cells showed higher sensitivity to both PAE and PEE as compared to MDA-MB-231 cells. LC_50_ values of both the extracts in PBMC and J774A cell line were >300 μg/ml (**Table 1**). Furthermore, microscopic visualization indicated a cytotoxic effect of the extracts upon these cancer cell lines (**Figures 1D–F**, **2D–F**). All these findings suggested that the fruit pulp extracts induced reduced viability of cells that is presumably caused by inhibiting cell proliferation and inducing cytotoxicity.

**Table 1:** Cytotoxic effect [LC_50_ (μg/ml)] of *Citrullus colocynthis* pulp extracts against two breast cancer (MDA-MB-231 and MCF-7), a cervical cancer (SiHa) cell lines, a human mononuclear cell PBMC and a macrophage cell J774A.

		LC50 (μg/ml) ± SEM				
		
		Cancer cell lines			Normal cell lines	
			
	Solvents (extraction)	MDA-MB-231	MCF-7	SiHa	PBMC	J774A
Colocynth fruit pulp	Ethanol	142 ± 16	105 ± 8.3	157 ± 4.3	>300	>300
	Acetone	140 ± 0.8	94 ± 6.4	121 ± 4.6	>300	>300


*Values represent mean ± SEM of three independent experiments.*

**FIGURE 3:**
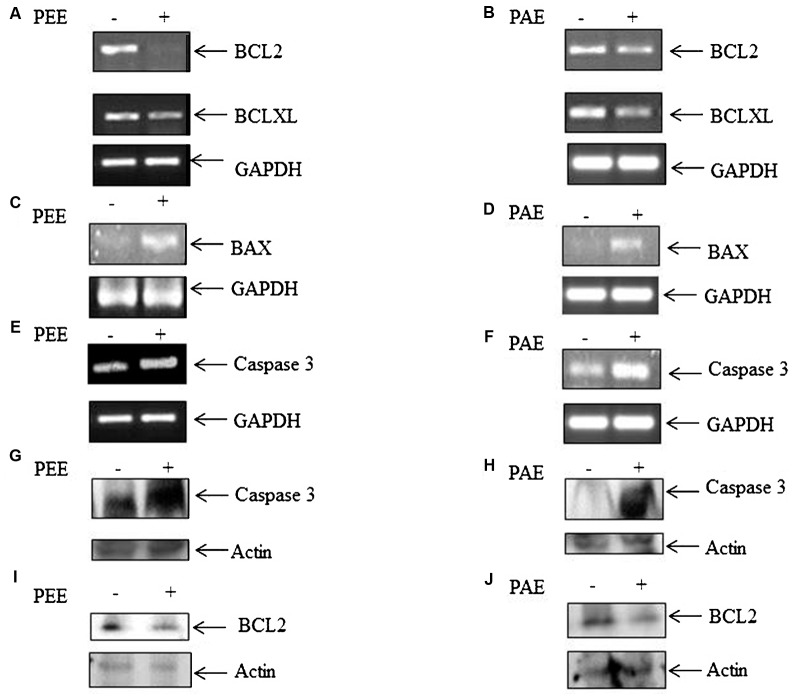
Effect of fruit pulp extracts on anti-apoptotic and apoptotic genes in MDA-MB-231 cells. **(A)** Cells were separately treated with pulp ethanol extract (PEE) **(A,C,E,G)** and pulp acetone extract (PAE) **(B,D,F,H)** for 24 h, and subsequently expression of anti-apoptotic genes **(A,B)** BCL2 and BCLXL, and pro-apoptotic genes BAX **(C,D)** and Caspase 3 **(E,F)** were measured by RT-PCR analysis, using total RNA isolated from cells with or without PEE and PAE treatment, and gene specific primers. Here, GAPDH was used as an internal loading control. Densitometric analysis data are given in Supplementary Figure S1. **(G,H)** Level of cleaved caspase 3 and **(I,J)** BCL-2 was measured by western blotting using equal amount of cellular protein isolated from cells with or without treatment with PEE or PAE. B-actin serves as an internal loading control.

To examine the influence of these pulp extracts on cell apoptosis, RT-PCR analysis was performed to compare the transcript levels of anti-apoptotic BCL2 and BCLXL and apoptotic BAX genes, using total RNA of MDA-MB-231 cells with or without treatment with ethanol and acetone extracts (50 μg/ml). Both PEE and PAE considerably impeded BCL2 and BCLXL expression with simultaneous upsurge in BAX expression (**Figures 3A–D** and Supplementary Figures S1A–C,E–G). Moreover, expression of apoptotic caspase 3 gene was augmented upon treatment with both PAE and PEE (**Figures 3E,F** and Supplementary Figures S1D,H). Furthermore, western blot results demonstrated increased level of cleaved caspase 3 (**Figures 3G,H**) with a concomitant decrease in the protein level of anti-apoptotic gene BCL2 (**Figures 3I,J**) for both ethanol and acetone extract treated cells compared to control. These observations led us to propose an apoptotic activity of fruit pulp extracts in breast cancer cells.

### Inhibitory Effect of Colocynth Pulp Extracts on Cancer Cell Migration

**FIGURE 4:**
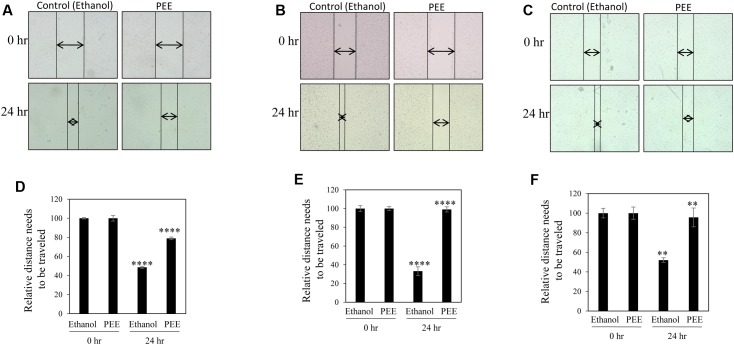
Pulp ethanol extract inhibited migration of breast and cervical cancer cells. Breast cancer MDA-MB-231 **(A)**, MCF-7 **(B)** and cervical cancer (SiHa) **(C)** cells were treated with pulp ethanol extract (PEE) for 24 h. Photos of cells at the starting time point and after 24 h of treatment were captured by inverted bright field microscope. The unfilled gap areas between two ends of the scratch were measured and subsequently plotted. Significant inhibition of cell migration was observed in case of PEE treated plates wells as compared to control in MDA-MB-231 **(D)**, MCF-7 **(E)** and SiHa **(F)** cancer cell lines. Percent inhibition by PEE after 24 h was calculated with the 0 h being the control. Values represent mean ± SE of triplicate measurements. In **(D)** ^∗∗∗∗^*p* < 0.0001; control (ethanol) 24 h vs. control (ethanol) 0 h and ^∗∗∗∗^*p* < 0.0001; PEE 24 h vs. control 24 h. In **(E)** ^∗∗∗∗^*p* < 0.0001; control (ethanol) 24 h vs. control (ethanol) 0 h and ^∗∗∗∗^*p* < 0.0001; PEE 24 h vs. control (ethanol) 24 h. In **(F)** ^∗∗^*p* < 0.01; control (ethanol) 24 h vs. control (ethanol) 0 h and ^∗∗^*p* < 0.01; PEE 24 h vs. control (ethanol) 24 h.

**FIGURE 5:**
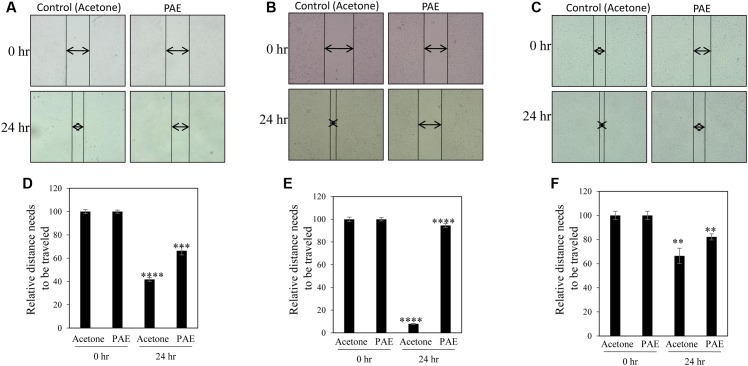
Pulp acetone extract inhibited migration of breast and cervical cancer cells. Breast cancer MDA-MB-231 **(A)**, MCF-7 **(B)** and cervical cancer (SiHa) **(C)** cells were treated with pulp acetone extract (PAE) for 24 h. Photos of cells at the starting timepoint and after 24 h of treatment were captured by inverted bright field microscope. The unfilled gap areas between two ends of the scratch were measured and subsequently plotted. Significant inhibition of cell migration was observed in case of PAE treated plates wells as compared to control in MDA-MB-231 **(D)**, MCF-7 **(E)** and SiHa **(F)** cancer cell lines. Percent inhibition by PAE after 24 h was calculated with the 0 h being the control. Values represent mean ± SE of triplicate measurements. In **(D)** ^∗∗∗∗^*p* < 0.0001; control (acetone) 24 h vs. control (acetone) 0 h and ^∗∗∗^*p* < 0.001; PAE 24 h vs. control (acetone) 24 h. In **(E)** ^∗∗∗∗^*p* < 0.0001; control (acetone) 24 h vs. control (acetone) 0 h and ^∗∗∗∗^*p* < 0.0001; PAE 24 h vs. control (acetone) 24 h. In **(F)** ^∗∗^*p* < 0.01; control (acetone) 24 h vs. control (acetone) 0 h. and ^∗∗^*p* < 0.01; PAE 24 h vs. control (acetone) 24 h.

A common problem faced by a large percentage of cancer patients is development of metastasis if cancers are not detected in early and/or not treated with a suitable therapy. Migration of cancer cells is a critical step in the metastatic cascade (Chaffer and Weinberg, 2011). Thus, we investigated the effect of pulp ethanol and acetone extracts on the migratory property of cancer cells by performing scratch assay. Scratches were made on cell monolayer by a small pipet tip, and subsequently, cells were incubated with or without pulp extracts. Microscopic photos indicated migration of cells to close the induced scratch after 24 h in control MDA-MB-231, MCF-7 and SiHa cells (**Figures 4A–C**, **5A–C**). Cells treated with both ethanol and acetone extracts (25 μg/ml) showed substantial prevention of cell migration as evident from the unfilled gap areas between the two ends of the scratches that were larger in cases of pulp extracts as compared to control. The data displayed in **Figures 4D–F**, **5D–F** denoted a significant inhibition of cell migration in response to treatment with both ethanol and acetone extracts.

### Effect of Colocynth Pulp Extracts on EMT

**FIGURE 6:**
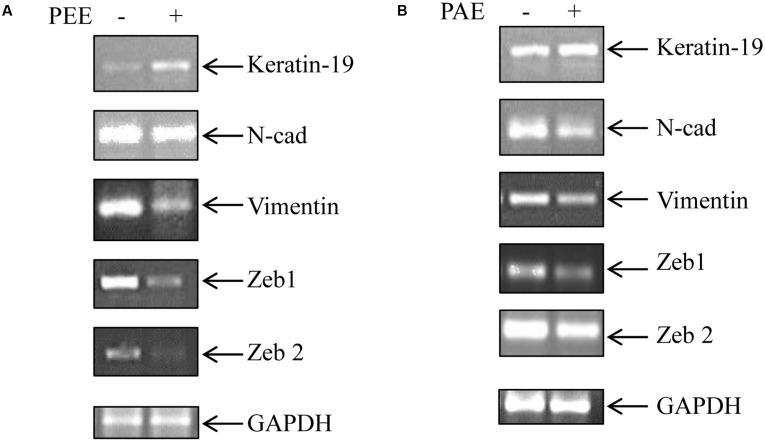
Effect of colocynth pulp extracts on expression of EMT associated genes in breast cancer cells. Breast cancer MDA-MB-231 cells were either treated with pulp ethanol extract (PEE) **(A)** or pulp acetone extract (PAE) **(B)** for 24 h, and subsequently the expression of EMT associated genes was measured by RT-PCR analysis using total RNA isolated from cells. GAPDH was used an internal loading control. Densitometric data is given in Supplementary Figure S2.

Transition from epithelial to mesenchymal phenotype is a critical step for metastasis (Chaffer and Weinberg, 2011). To examine the effect of fruit pulp extracts on prevention of EMT of metastatic MDA-MB-231 cells, we analyzed mRNA levels of several EMT markers by RT-PCR. Interestingly, we observed a decline in expression of mesenchymal markers, Vimentin and *N*-cadherin, with a concurrent increase in the expression of epithelial marker keratin-19 in treated samples (**Figures 6A,B** and Supplementary Figures S2A–C,F–H). Moreover, both PEE and PAE treatment strongly inhibited expression of the mesenchymal phenotype inducing transcription factors, Zeb1 and Zeb2 (**Figures 6A,B** and Supplementary Figures S2D,E,I,J). These observations clearly indicate a restraining impact of these pulp extracts on EMT of breast cancer cells.

### Colocynth Pulp Extracts Inhibited Stemness Property of Breast Cancer Cells

**FIGURE 7:**
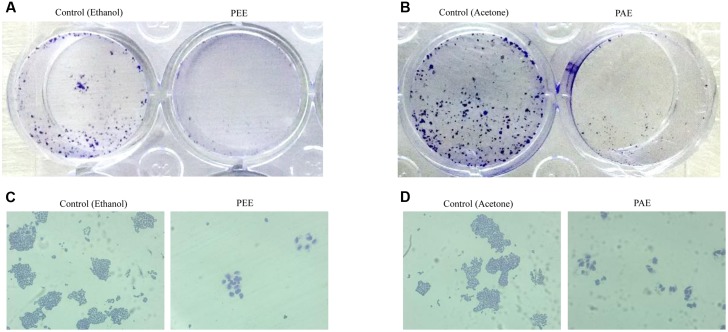
Effect of colocynth pulp extracts on colony formation of breast cancer cell. MDA-MB-231 cells were treated with pulp ethanol extract (PEE) **(A,C)** and pulp acetone extract **(B,D)**. After 5 days of cell seeding, photos of the wells were taken by a camera **(A,B)**, and colonies were also visualized by inverted bright field microscope **(C,D)**.

**FIGURE 8:**
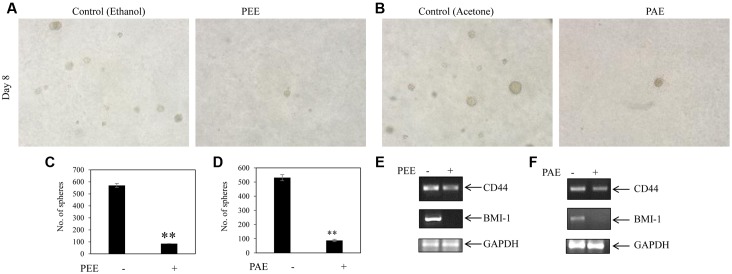
Inhibitory activity of colocynth pulp extract on spheroid formation and stemness of breast cancer cell. **(A)** Soft agar assay was performed on MDA-MB-231 cells by treating cells with pulp ethanol extract (PEE; **A,C**) and pulp acetone extract (PAE; **B,D**) and allowing cells to grow for 8 days. Spheroid colonies were visualized by inverted bright field microscope **(A,B)**. Number of spheroids of all plates was counted and analyzed with respect to control plates **(C,D)**, in **(C)** ^∗∗^*p* < 0.01; PEE vs. control (ethanol) after 8 days, and in **(D)** ^∗∗^*p* < 0.01; PAE vs. control (acetone) after 8 days. **(E,F)** MDA-MB-231 cells were treated with PEE **(E)** and PAE **(F)** for 24 h and subsequently, expression of CD44 and BMI-1 was compared by RT-PCR analysis using total RNA isolated from cells and CD44 and BMI-1 gene specific primers. GAPDH was used as an internal loading control. Densitometric data is given in Supplementary Figure S3.

Accumulating evidences suggest that cancers develop from cancer initiating cells or cancer stem cells (CSCs) (Reya et al., 2001; Singh and Settleman, 2010). Although CSCs comprise of a small percentage of the tumor population, their resistance to contemporary chemo- and radiation- therapy is a leading cause for tumor recurrence and metastasis (Li et al., 2007; Singh and Settleman, 2010). Thus, we investigated the effect of colocynth pulp extracts on the stemness property of metastatic MDA-MB-231 cells by colony formation assay. In agreement with previous observations, the number of colony formed was markedly reduced when cells were treated with pulp extracts (25 μg/ml) as compared to control, indicating an inhibitory role of both PEE and PAE on cancer stemness (**Figures 7A–D**). To further support these results, we carried out soft agar sphere formation assay. Microscopic visualization documented formation of a great number of spheres with large size after 8 days of cell culture in the control plates. The number as well as the size of such spheres was, however, greatly diminished when cells were incubated with both PAE and PEE (25 μg/ml) as compared to control (**Figures 8A–D**). These results show that fruit pulp extracts might attenuate stemness property of cancer cells. To verify these findings, we further examined the effect of these pulp extracts on the expression of stemness associated genes CD44 and BMI-1. RT-PCR analysis documented that transcript levels of both CD44 and BMI-1 genes (Al-Hajj et al., 2003; Liu et al., 2006) were significantly lowered in MDA-MB-231 cells when treated with both PAE and PEE as compared to control (**Figures 8E,F** and Supplementary Figures S3A–D). Together, these data clearly suggested that fruit pulp extracts (PEE and PAE) obliterate the stemness property of breast cancer cells.

## Discussion

Considering the current status of diagnostic and treatment options available, cancer associated deaths are on the rise. In India, carcinoma of the breast and cervix contributes to 50% of total cancer incidences^2^. However, surgical removal, radiation-, chemo-, endocrine-, and other targeted- therapies are continuously being improvised to improve the overall survival and quality of life of cancer patients. Although modern treatment modalities are capable of taking care of the primary tumors, tumor recurrence and metastasis are the major limitations in the limelight at present. Furthermore, chemotherapeutic drugs often show significant off-target effects along with systemic toxicity, such as cardiovascular, liver and nephrotoxicity (King and Perry, 2001; Schimmel et al., 2004). Thus, finding novel anticancer drugs, which prevent both tumor growth and metastasis while circumventing the known side effects, is an active area of research. In this area, application of natural products like secondary metabolites with effective tumor suppressive ability has shown promise in recent years as they are found to be effective even at sufficiently lower doses and due to their safety profile (Koehn and Carter, 2005).

Thus, this study was aimed at exploring the anti-cancer properties of extracts from *Citrullus colocynth* plant of the cucurbitaceae family that has been used as a traditional medicine to control diabetes (Zaini et al., 2011; Shi et al., 2014). Reports about the nutritional and medicinal values of this plant and presence of many bioactive molecules had initially attracted our attention (Sawaya et al., 1983, 1986; Asyaz et al., 2010; Marzouk et al., 2010; Hussain et al., 2013, 2014; Jeon and Lee, 2014; Chawech et al., 2017). Moreover, Tannin-Spitz et al. (2007) found that an isolate (cucurbitacin glucosides) extracted from the leaves of this plant exhibits apoptotic activity in cancer cells. Nevertheless, no detailed study was done regarding the anti-cancer effects of this plant extract. Our study found that both ethanol and acetone extracts of fruit pulp of the colocynth plant showed a significant inhibition of cell viability of various cancer cell lines, including breast cancer MCF-7 and MDA-MB-231, and cervical cancer SiHa cell lines (**Figures 1**, **2** and **Table 1**). Correspondingly, both extracts had reverse cytotoxic effect on mononuclear cells PBMC and macrophage cell line J774A. Breast cancer MCF-7 cells showed more sensitivity to both ethanol and acetone pulp extracts as compared to other cancer cells. This was in agreement with previous reports that too claim higher sensitivity of MCF-7 cells to colocynth fruit extract at relatively lower concentrations (Mukherjee and Patil, 2012). The probable explanation may be either use of alkaloid rich extracts or longer exposure in MCF-7 cells (Mukherjee and Patil, 2012). Our study also found that both ethanol and acetone extracts exhibited synergistic potential with the chemotherapeutic drug doxorubicin to inhibit viability in the breast cancer cells (Supplementary Figure S4).

Moreover, on molecular analysis, we found suppression of transcripts of anti-apoptotic genes BCL2 and BCLXL, and an induction of apoptotic gene BAX and caspase 3 mRNA in metastatic breast cancer MDA-MB-231 cells following treatment with ethanol and acetone pulp extracts (**Figure 3**). In addition, these pulp extracts displayed a higher level of cleaved caspase 3 protein and a lower level of BCL-2 protein as compared to control, implying an enhancement of active caspase 3 upon treatment with these extracts (**Figure 3**). Thus, this study indicated that colocynth fruit pulp extracts exhibit an anticancer potential presumably by promoting apoptosis.

As stated earlier, metastasis remains one of the major threats to cancer patients. However, no study has yet been conducted to investigate the anti-metastatic potential of this plant extract. Our study indicated, for the first time, a preventive role of these pulp extracts in cancer cell migration, a key step in the metastatic process (**Figures 4**, **5**) (Chaffer and Weinberg, 2011). These pulp extracts also block EMT (Chaffer and Weinberg, 2011), another vital step in the metastatic cascade, since both PEE and PAE treatment significantly enhanced epithelial gene expression with simultaneous inhibition of mesenchymal marker expression in breast cancer MDA-MB-231 cells (**Figure 6**). Additionally, treatment of cancer cells with these ethanol and acetone pulp extracts showed a noticeable reduction in colony and sphere formation with concomitant inhibition of cancer stemness associated genes CD44 and BMI-1 at their transcript levels, suggesting a preventive role of these pulp extracts in cancer stemness (Monteiro and Fodde, 2010), a pivotal property responsible for metastasis, therapy resistance and tumor recurrence (**Figures 7**, **8**).

All these findings summarized a role of the ethanol and acetone fruit pulp extracts of colocynth in prevention of cell proliferation, induction of cell apoptosis, prevention of cell migration, reduction of EMT, and inhibition of cancer stemness property in breast cancer cells.

However, further study is required to identify the bioactive molecule(s) present in fruit pulp, liable for the observed anticancer potential. Subsequent GC-MS/MS analysis of both the extracts revealed about fifty compounds (Supplementary Figures S5A,B and Tables S2, S3) out of which, we analyzed the top 20 compounds on the basis of abundance in the extracts (**Table 2**). More in-depth study narrowed the list to those compounds that are novel to the extracts, with no previous reports of biological study (**Table 3**). We have also noticed that some compounds are common both in ethanolic and acetone extracts. Thus, purification and more detailed investigation of these compounds will enable identification of the potent constituents in the extracts that are accountable for retarded cell growth and metastasis of cancer cells. Further mechanistic studies will prove extremely useful for their utility in future therapeutic interventions.

**Table 2:** Top 20 compounds isolated from GC-MS/MS analysis of ethanol and acetone crude extract.

S. No	RT	Compound name	Area %	Molecular formula	Molecular weight	Biological study
**Ethanol**						
1	20.64	Naphtho[1,2-d]oxazol-2(1H)-one, 3a,4,5,9b-tetrahydro-3a,5,5,9b-tetramethyl-1-(phenylmethyl)-	31.32	C22H25NO2	335	Not reported
2	21.64	1,5-Diphenylhex-3-ene	14.72	C18H20	236	Anti-microbial
3	28.56	Benzenesulfonic acid, 4-methoxy-, [(4-methylphenyl)sulfonyl]methyl ester	5.56	C15H16O6S2	356	Not reported
4	24.6	Heptyl methyl methylphosphonate	4.55	C8H19O3P	194	Not reported
5	7.58	DL-4,5-Octanediol	3.28	C8H18O2	146	Not reported
6	16.99	Oxalic acid, allyl dodecyl ester	3.23	C17H30O4	298	Yes
7	21.97	10-Pentadecen-5-yn-1-ol, (E)-	3.22	C15H26O	222	Not reported
8	29.33	(E,E,E)-(5-Phenylsulfonylgeranyl)geraniol	2.71	C26H38O3S	430	Not reported
9	18.71	*cis,cis,cis*-7,10,13-Hexadecatrienal	2.46	C16H26O	234	Yes, anti-microbial
10	26.46	2-Isopropyl-5-methylcyclohexyl 3-(1-(4-chlorophenyl)-3-oxobutyl)-coumarin-4-yl carbonate	2.26	C30H33ClO6	524	Not reported
11	22.52	Cyclopenta[c]pyran-1,3-dione, 4,4a,5,6-tetrahydro-4,7-dimethyl-	2	C10H12O3	180	Yes, anti-insecticidal
12	26.51	2-Isopropyl-5-methylcyclohexyl 3-(1-(4-chlorophenyl)-3-oxobutyl)-coumarin-4-yl carbonate	1.98	C30H33CLO6	524	Not reported
13	13.04	2,2-Dimethyl-propyl 2,2-dimethyl-propanesulfinyl sulfone	1.7	C10H22O3S2	254	Yes
14	14.43	á-l-Arabinopyranoside, methyl	1.59	C6H12O5	164	Yes
15	8.78	Cycloheptatrienylium, iodide	1.46	C7H7I	218	Not reported
16	18.62	Cyclohexanemethyl propanoate	1.41	C10H18O2	170	Yes
17	20.47	2,2-Dimethyl-propyl 2,2-dimethyl-propanesulfinyl sulfone	1.36	C10H22O3S2	254	Yes
18	23.01	Benzenesulfonic acid, 4-methoxy-, [(4-methylphenyl)sulfonyl]methyl ester	1.33	C15H16O6S2	356	Not reported
19	28.31	Dimethylsilyl tert-buthylperoxide	1.24	C6H16O2Si	148	Not reported
20	23.9	Heptyl methyl methylphosphonate	1.02	C9H21O3P	208	Not reported
**Acetone**						
1	18.91	Oxalic acid, cyclobutyl octadecyl ester	24.73	C24H44O4	396	Yes
2	21.02	9,12,15-Octadecatrienal	16.98	C18H30O	262	Yes
3	14	2,2-Dimethyl-propyl 2,2-dimethyl-propanesulfinyl sulfone	14.46	C10H22O3S2	254	Yes
4	20.91	Z-4-Dodecenol	10.32	C12H24O	184	Yes
5	28.95	Carbonic acid, 3-hexenyl methyl ester, (Z)-	5.24	C8H14O3	158	Yes
6	23.61	Naphtho[1,2-d]oxazol-2(1H)-one, 3a,4,5,9b-tetrahydro-3a,5,5,9b-tetramethyl-1-(phenylmethyl)-	4.79	C22H25O2	335	Not reported
7	24.74	17-Octadecene-9,11-diynoic acid, 8-hydroxy-, methyl ester	3.36	C19H28O3	304	Not reported
8	21.13	1-Cyclohexylnonene	2.84	C15H28	208	Not reported
9	8.71	Cycloheptatrienylium, iodide	1.24	C7H7I	218	Not reported
10	24.64	Cyclohexanemethyl propanoate	1.2	C10H18O2	170	Yes
11	10.1	3-Selenetanol, 3-(4-methoxyphenyl)-	1.12	C10H12O2Se	244	Not reported
12	7.71	Cyclobutanecarboxylic acid, but-3-yn-2-yl ester	1.1	C10H14O2	166	Not reported
13	23.85	1-Benzylbenzimidazole 3-oxide	0.85	C14H12N2O	224	Yes
14	25.86	Sucrose	0.75	C12H22O11	342	Yes
15	29.4	9-Azabicyclo[6.1.0]nonane, 9,9′-azobis-, [1à,8à,9[E(1’R^∗^,8’S^∗^)]]-	0.75	C16H28N4	276	Not reported
16	7.77	N,N′-Bis(2,6-dimethyl-6-nitrosohept-2-en-4-one)	0.7	C18H30N2O4	338	Not reported
17	15.63	Z-2-Dodecenol	0.69	C12H24O	184	Yes
18	27.46	2-(4-Bromobutyl)-furan	0.59	C8H11BrO	202	Not reported
19	18.58	2,2-Dimethyl-propyl 2,2-dimethyl-propanesulfinyl sulfone	0.58	C10H22OS2	254	Yes
20	24.84	5,10-Pentadecadiyn-1-ol	0.55	C15H24O	220	Yes


*Compounds were selected on the basis of top twenty peak area extracted from the ethanol crude extract of fruit from the plant *Citrullus colocynthis*. Interpretation of the compound activity was done, and thus a comparative analysis is given with their reported molecular formula.*

**Table 3:** List of extracted novel compounds.

S. No	RT	Compound name	Area %	Molecular formula	Biological study
**Ethanol plant extract**					
1	23.84	Phosphonic acid, methyl-, 2,2-dimethylcyclohexyl methyl ester	0.73	C10H21O3P	NA
2	16.34	2-Trimethylsiloxy-6-hexadecenoic acid, methyl ester	0.25	C20H40O3Si	NA
3	21.05	Manganese(1+), dicarbonyl[(1,2,3,4,5-ü)-1-methyl-2,4-cyclopentadienyl]nitrosyl-	0.24	C8H8MnNO3	NA

1	20.64	Naphtho[1,2-d]oxazol-2(1H)-one, 3a,4,5,9b-tetrahydro-3a,5,5,9b-tetramethyl-1-(phenylmethyl)-	31.32	C22H25NO2	NA
2	21.64	2-Cyclohexene-6-carboxylic acid, 3-methyl-1-(5-oxo-3,4-dihydro-2H-pyrrolyl)-, ethyl ester	14.72	C14H21NO3	NA
3	28.56	Naphtho[1,2-d]oxazol-2(1H)-one, 3a,4,5,9b-tetrahydro-3a,5,5,9b-tetramethyl-1-(phenylmethyl)-	5.56	C22H25NO2	NA
4	18.71	Acrylic acid, 5,7-octadien-1-yl ester	2.46	C11H16O2	NA
5	26.46	2-Isopropyl-5-methylcyclohexyl 3-(1-(4-chlorophenyl)-3-oxobutyl)-coumarin-4-yl carbonate	2.26	C30H33ClO6	NA
6	26.51	4-Chloro-4-methylhexane-2,3-dione	1.98	C7H11ClO2	NA
7	29.1	Cyanomethyl 2-chloroethyl disulfide	0.26	C4H6ClNS2	NA
8	9.83	2-Methyl-2-(methoxy-ethoxy)amino-propan-1-ol	0.2	C7H17NO3	NA
9	25.25	5-[2-(1,3-Dioxolan-2-yl)-ethyl]-2-methyl-1-cyclopentene-1-carboxaldehyde	0.13	C12H18O3	NA

**Acetone plant extract**					
1	7.36	Propanamide, 3-(3,4-dimethylphenylsulfonyl)-	0.31	C11H15NO3S	NA
2	7.77	Cyclobutanecarboxylic acid, 4-nitrophenyl ester	0.7	C11H11NO4	NA
3	26.12	Bicyclo[3.2.1]octane, 2-bromo-4-iodo-	0.22	C8H12BrI	NA

1	23.61	Naphtho[1,2-d]oxazol-2(1H)-one, 3a,4,5,9b-tetrahydro-3a,5,5,9b-tetramethyl-1-(phenylmethyl)-	4.79	C22H25NO2	NA
2	24.74	4,5-Dihydroxy-6-hydroxymethyl-oxepan-3-one	3.36	C7H12O5	NA
3	21.13	2-Methyl-2-chloro-3-nitroso-4-cyclohexyloxy-butane	2.84	C11H20ClNO2	NA
4	7.71	Cyclobutanecarboxylic acid, but-3-yn-2-yl ester	1.1	C10H14O2	NA
5	7.71	Cyclobutanecarboxylic acid, pent-2-en-4-ynyl ester	1.1	C10H12O2	NA
6	25.86	6-Chloro-2,2,9,9-tetramethyl-3,7-decadiyn-5-ol	0.75	C14H21ClO	NA
7	7.77	Cyclobutanecarboxylic acid, but-3-yn-2-yl ester	0.7	C10H14O2	NA
8	26.43	5-[2-(1,3-Dioxolan-2-yl)-ethyl]-2-methyl-1-cyclopentene-1-carboxaldehyde	0.4	C12H18O3	NA
9	24.22	2-Bromoethyl vinyl sulfide	0.15	C4H7BrS	NA
10	27.59	*N*-Difluorophosphoxy -*O*-trimethylsilylhydroxylamine	0.15	C3H10F2NO2PSi	NA


*Compounds were isolated by GC-MS/MS analysis from ethanol and acetone pulp extract and tabulated on the basis of non-reported data.*

In brief, using cell culture based *in vitro* study and GC-MS/MS analysis, we report, for the first time, some novel compounds in the fruit pulp extracts of colocynth plant, that show promising potential as anti-cancer therapeutics for their tumor suppressive properties.

## Author Contributions

KC, AS, SK, and GG have substantial contribution in performing experiments, analyzing data, and interpreting results. KC has substantial involvement in drafting manuscript. AN has significant contribution in analyzing data and editing the manuscript. CM has formulated and supervised the work, and written the manuscript. All authors approved the final version of the manuscript and agree to be accountable for the content of this work.

## Conflict of Interest Statement

The authors declare that the research was conducted in the absence of any commercial or financial relationships that could be construed as a potential conflict of interest.

## Footnotes

1 breastcancerindia.net

2 breastcancerindia.net
