# Supplementary material for: Colocynth Extracts Prevent Epithelial to Mesenchymal Transition and Stemness of Breast Cancer Cells
Source: Front Pharmacol. 2017 Sep 5;8:593. doi: 10.3389/fphar.2017.00593 (PMC5591978; doi:10.3389/fphar.2017.00593)
Supplement: Supplementary file 1 [file Table_1.PDF]

Supplementary Table S1. Sequence details of human primers used in PCR reactions

Gene	Forward primer	Reverse primer
BCL2	GTGAACTGGGGGAGGATTGT	GGAGAAATCAAACAGAGGCC
BCLXL	ACCCCAGGGACAGCATATCA	TGCGATCCGACTCACCAATA
BAX	GGGTGGTTGGGTGAGACTC	AGACACGTAAGGAAAACGCATTA
Caspase-3	ACATGGCGTGTCATAAAATACC	CACAAAGCGACTGGATGAAC
Vimentin	TCCAGCAGCTTCCTGTAGGT	CCCTCACCTGTGAAGTGGAT
Keratin-19	GCGGGACAAGATTCTTGGTG	CTTCAGGCCTTCGATCTGCAT
N-cadherin	CGAATGGATGAAAGACCCATCC	GGAGCCACTGCCTTCATAGTCAA
Zeb 1	TGCACTGAGTGTGGAAAAGC	TGGTGATGCTGAAAGAGACG
Zeb 2	CGCTTGACATCACTGAAGGA	CTTGCCACACTCTGTGCATT
CD44	CGGACACCATGGACAAGTTT	GAAAGCCTTGCAGAGGTCAG
BMI1	GTCCAAGTTCACAAGACCAGACC	ACAGTCATTGCTGCTGGGCATCG
GAPDH	ACCCAGAAGACTGTGGATGG	CACGTTGGCAGTGGGGACAC

Supplementary Table S2. Total GC-MS/MS analysis of crude ethanol pulp extract fraction of fruit from the plant *Citrullus colocynth* (C.C.)

S.No	RT	Compound Name	Area %
1	6.83	Cyclohexane-1,3-dione, 2-(2-hydroxyethylaminomethylene)-5,5-dimethyl-	0.98
2	7.58	DL-4,5-Octanediol	3.28
3	8.78	Cycloheptatrienylium, iodide	1.46
4	9.83	Methyl 4-ketohex-5-enoate	0.2
5	10.14	BUTYL ISOBUTYL ISOBUTAL	0.29
6	10.98	3,4-Hexanediol, 3,4-dimethyl-	0.15
7	12.03	2,6,10,14-Tetramethylpentadecan-2-ol	0.19
8	13.04	2,2-Dimethyl-propyl 2,2-dimethyl-propanesulfinyl sulfone	1.7
9	13.99	à-D-Xylofuranoside, methyl 5-O-methyl-	0.15
10	14.43	á-l-Arabinopyranoside, methyl	1.59
11	14.79	5-Thio-d-glucopyranose	0.15
12	14.92	2-Isopropyl-5,6-dimethyl-1,3,2-oxathiaborinane	0.52
13	15.02	2-Heptadecenal	0.24
14	15.82	5-Thio-d-glucopyranose	0.18
15	16.34	Methyl 3-methyl-3-(methoxy-ethoxy)amino-butanoate	0.25
16	16.99	Oxalic acid, allyl dodecyl ester	3.23
17	18.62	Cyclohexanemethyl propanoate	1.41
18	18.71	cis,cis,cis-7,10,13-Hexadecatrienal	2.46
19	18.78	Cyclohexanemethyl propanoate	0.87
20	19.67	Tetradecane, 1-iodo-	0.49
21	20.47	2,2-Dimethyl-propyl 2,2-dimethyl-propanesulfinyl sulfone	1.36
22	20.64	Naphtho[1,2-d]oxazol-2(1H)-one, 3a,4,5,9b-tetrahydro-3a,5,5,9b-tetramethyl-1-(phenylmethyl)-	31.32
23	20.9	5,10-Pentadecadiyne, 1-chloro-	0.24
24	21.05	Manganese(1+), dicarbonyl[(1,2,3,4,5-ü)-1-methyl-2,4-cyclopentadienyl]nitrosyl-	0.24
25	21.24	Dichloroacetic acid, 2,2-dimethylpropyl ester	0.95
26	21.64	1,5-Diphenylhex-3-ene	14.72

27	21.97	10-Pentadecen-5-yn-1-ol, (E)-	3.22
28	22.22	Benzene, 1,1'-[1,4-butanediylbis(oxymethylene)]bis-	0.31
29	22.52	Cyclopenta[c]pyran-1,3-dione, 4,4a,5,6-tetrahydro-4,7-dimethyl-	2
30	22.75	1,3-Dioxolane, 2-(1-phenylethyl)-	0.21
31	22.79	3-Hexen-1-ol, 2-ethyl-	0.28
32	23.01	Benzenesulfonic acid, 4-methoxy-, [(4-methylphenyl)sulfonyl]methyl ester	1.33
33	23.69	2-Propene(dithioic) acid, 3-(4-chlorophenyl)-3-hydroxy-	0.17
34	23.84	5,10-Pentadecadien-1-ol, (Z,Z)-	0.73
35	23.9	Heptyl methyl methylphosphonate	1.02
36	24.18	7-Acetyl-2-hydroxy-2-methyl-5-isopropylbicyclo[4.3.0]nonane	0.29
37	24.6	Heptyl methyl methylphosphonate	4.55
38	25.18	Benzoic acid, 3-methoxy-4-[(methylsulfonyl)oxy]-	0.23
39	25.25	2,6,10-Dodecatrien-1-ol, 3,7,11-trimethyl-9-(phenylsulfonyl)-, (E,E)-	0.13
40	25.57	2,4-Pentanedione, 1,1,1-trifluoro-5-phenyl-	0.37
41	25.66	Carbonic acid, 3-hexenyl methyl ester, (Z)-	0.96
42	25.82	3-Hexene, 1-(1-ethoxyethoxy)-, (Z)-	0.81
43	26.46	2-Isopropyl-5-methylcyclohexyl 3-(1-(4-chlorophenyl)-3-oxobutyl)-coumarin-4-yl carbonate	2.26
44	26.51	2-Isopropyl-5-methylcyclohexyl 3-(1-(4-chlorophenyl)-3-oxobutyl)-coumarin-4-yl carbonate	1.98
45	27.53	2,6,10-Dodecatrien-1-ol, 3,7,11-trimethyl-9-(phenylsulfonyl)-, (E,E)-	0.18
46	28.26	1,2-Benzenedicarboperoxoic acid, bis(1,1-dimethylethyl) ester	0.58
47	28.31	Dimethylsilyl tert-buthylperoxide	1.24
48	28.56	Benzenesulfonic acid, 4-methoxy-, [(4-methylphenyl)sulfonyl]methyl ester	5.56
49	29.1	3-Octanol	0.26
50	29.33	(E,E,E)-(5-Phenylsulfonylgeranyl)geraniol	2.71

50 compounds were found from the ethanol pulp extract ranging on the basis of retention time (R.T).

Supplementary Table S3. GC-MS/MS analysis of crude acetone pulp extract of the fruit in the plant *Citrullus colocynthis*.

S.No	RT	Compound Name	Area %
1	7.36	Thiophene-2-ol, benzoate	0.31
2	7.62	Octadecane, 2-methyl-	0.3
3	7.71	Cyclobutanecarboxylic acid, but-3-yn-2-yl ester	1.1
4	7.77	N,N'-Bis(2,6-dimethyl-6-nitrosohept-2-en-4-one)	0.7
5	8.71	Cycloheptatrienylium, iodide	1.24
6	10.1	3-Selenetanol, 3-(4-methoxyphenyl)-	1.12
7	10.34	5-Thio-d-glucopyranose	0.29
8	12.35	3,5-Dimethyl-4-octanone	0.37
9	13.08	N-Benzyloxy-2-isopropoxycarbonylazetidine	0.25
10	14	2,2-Dimethyl-propyl 2,2-dimethyl-propanesulfinyl sulfone	14.46
11	14.55	à-D-Xylofuranoside, methyl	0.15
12	15.02	2-[1,2-Dihydroxyethyl]-9-[á-d-ribofuranosyl]hypoxanthine	0.14
13	15.44	5-Thio-d-glucopyranose	0.11
14	15.63	Z-2-Dodecenol	0.69
15	16.22	S-1-Propenylpropanethiosulfonate	0.12
16	16.44	Sucrose	0.15
17	16.74	Carbamic acid, methylnitroso-, ethyl ester	0.21
18	17	Phosphoric acid, ethenyl dimethyl ester	0.17
19	17.11	Bicyclo[4.1.0]heptane, 7-pentyl-	0.4
20	17.68	2,2-Dimethyl-propyl 2,2-dimethyl-propanesulfinyl sulfone	0.54
21	18.58	2,2-Dimethyl-propyl 2,2-dimethyl-propanesulfinyl sulfone	0.58
22	18.91	Oxalic acid, cyclobutyl octadecyl ester	24.73
23	20.02	cis-2-Nitro-4-t-butylcyclohexanone	0.4
24	20.48	Ethanethioic acid, S-(2-methylbutyl) ester	0.47
25	20.91	Z-4-Dodecenol	10.32
26	21.02	9,12,15-Octadecatrienal	16.98
27	21.13	1-Cyclohexylnonene	2.84
28	21.5	Propionic acid, 3-(allylthio)-, propyl ester	0.14

	4		
29	23.61	Naphtho[1,2-d]oxazol-2(1H)-one, 3a,4,5,9b-tetrahydro-3a,5,5,9b-tetramethyl-1-(phenylmethyl)-	4.79
30	23.85	1-Benzylbenzimidazole 3-oxide	0.85
31	23.98	D-arabino-Hex-1-enitol, 1,5-anhydro-2-deoxy-	0.18
32	24.22	2(5H)-Furanone, 3-chloro-5-((dimethylamino)methyl)-4,5-dimethyl-	0.15
33	24.64	Cyclohexanemethyl propanoate	1.2
34	24.74	17-Octadecene-9,11-diynoic acid, 8-hydroxy-, methyl ester	3.36
35	24.84	5,10-Pentadecadiyn-1-ol	0.55
36	25.06	Propanoic acid, pentyl ester	0.22
37	25.35	4-Cyclohexene-1,2-dicarboxylic acid, 4-chloro-, bis(trimethylsilyl) ester	0.12
38	25.56	meso-2,5-Dimethyl-3,4-hexanediol	0.25
39	25.86	Sucrose	0.75
40	26.01	à-D-Xylofuranoside, methyl	0.28
41	26.12	Tricyclo[2.2.1.0(1,4)]heptan-2-one, 6-nitro-	0.22
42	26.43	5-[2-(1,3-Dioxolan-2-yl)-ethyl]-2-methyl-1-cyclopentene-1-carboxaldehyde	0.4
43	26.55	Parthenolide	0.27
44	27.46	2-(4-Bromobutyl)-furan	0.59
45	27.59	N-Difluorophosphoxy-O-trimethylsilylhydroxylamine	0.15
46	27.84	1,1,1,3,3,3-Hexafluoropropan-2-one, o-trimethysilyloxime	0.19
47	27.94	Cyclohexanol, 4-ethenyl-4-methyl-3-(1-methylethenyl)-, (1à,3à,4á)-	0.11
48	28.07	à-D-Xylofuranoside, methyl	0.11
49	28.95	Carbonic acid, 3-hexenyl methyl ester, (Z)-	5.24
50	29.4	9-Azabicyclo[6.1.0]nonane, 9,9'-azobis-, [1à,8à,9[E(1'R*,8'S*)]]-	0.75

GC-MS analysis of the acetone pulp fractions also yielded 50 compounds with retention time (RT) ranging from 7.36 to 29.40.
