# Supplementary material for: Colocynth Extracts Prevent Epithelial to Mesenchymal Transition and Stemness of Breast Cancer Cells
Source: Front Pharmacol. 2017 Sep 5;8:593. doi: 10.3389/fphar.2017.00593 (PMC5591978; doi:10.3389/fphar.2017.00593)
Supplement: Supplementary file 2 [file Image_1.pdf]

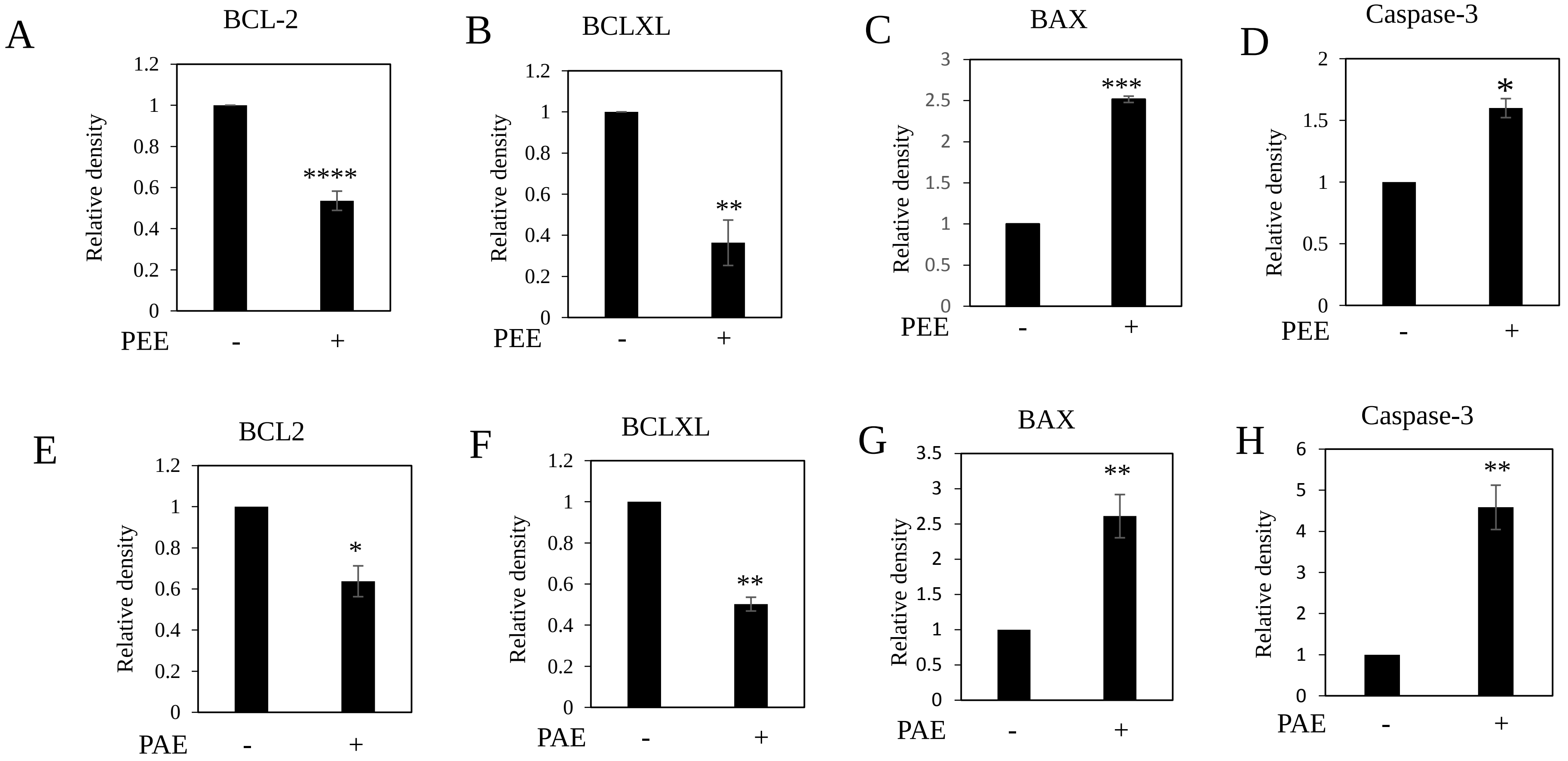

Supplementary Figure S1: Density analysis of PCR products (Figure 3) of anti-apoptotic and apoptotic genes . Densitometry analysis ((**A** –H) (ratio of respective gene/GAPDH) of these PCR bands was shown for BCL2 (A), BCLXL (B), BAX (C), caspase 3 (D) in case of PEE treated samples, and for BCL2 (E), BCLXL (F), BAX (G), caspase 3 (H) in case of PAE treated samples. All the density analysis is normalized with respect to GAPDH (loading control). Here, * $p<0.05$, ** $p<0.01$, *** $p<0.001$, and **** $p<0.0001$; treated with extract vs. control.

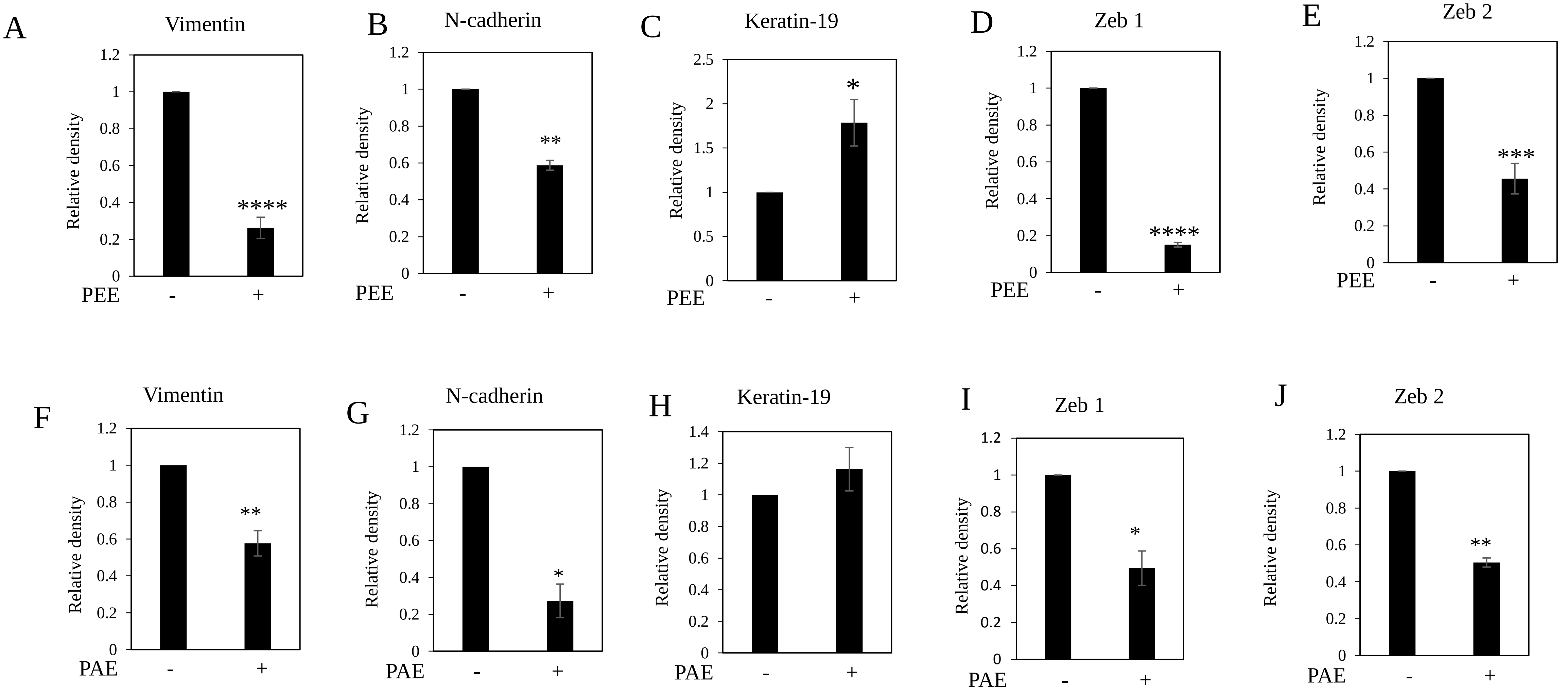

Supplementary Figure S2: Density analysis of PCR products (Figure 6) of EMT genes. (A-J) Densitometry analysis (ratio of respective gene/GAPDH) of these PCR bands was shown for Vimentin (A), N-Cadherin (B), Keratin-19 (C), Zeb 1(D), Zeb 2 (E) in case of PEE treated samples, for Vimentin (F), N-Cadherin (G), Keratin-19 (H), Zeb 1(I), Zeb 2 (J) in case of PAE treated samples. Here, GAPDH was used as an internal loading control. All the density analysis is normalized with respect to GAPDH . Here, * $p<0.05$, ** $p<0.01$, *** $p<0.001$, and **** $p<0.0001$; treated with extract vs. control.

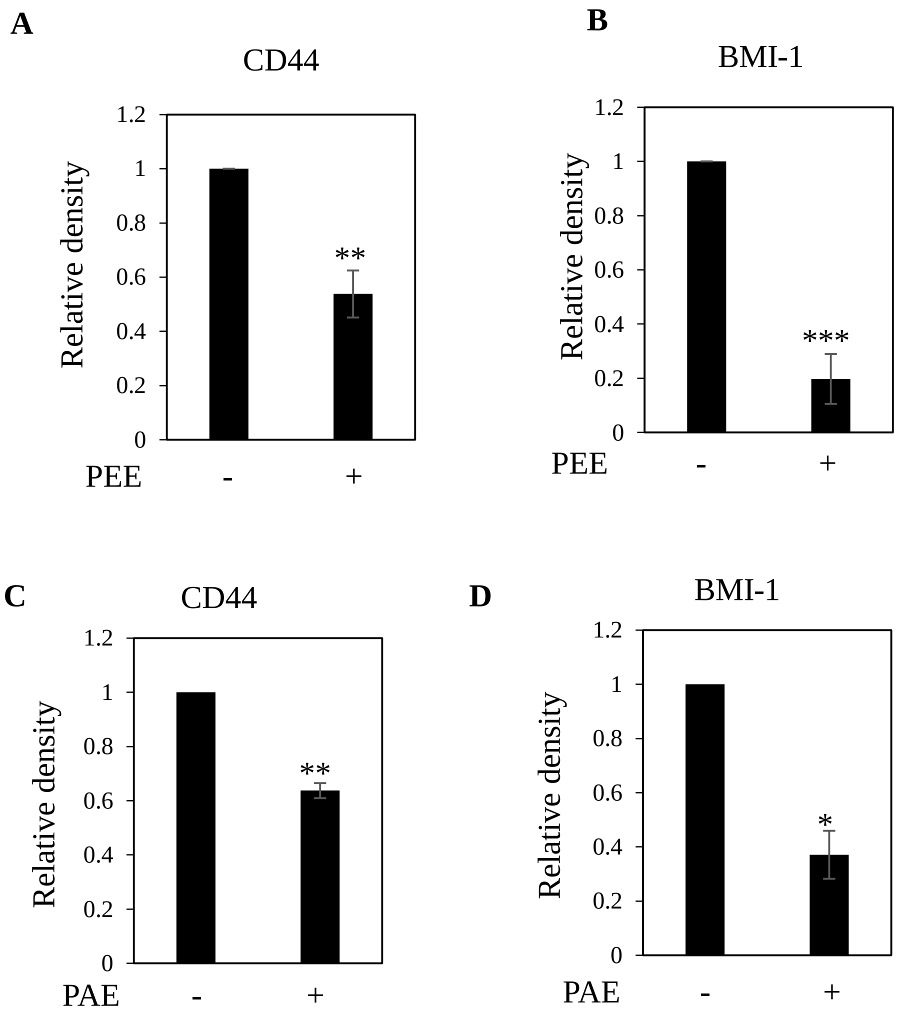

Supplementary Figure S3: Density analysis of PCR products (Figure 8) of stemness associated genes. (A-D) Densitometry analysis (ratio of respective gene/GAPDH) of these PCR bands was shown for CD44 (A), BMI-1 (B) in case of PEE treated samples, for CD44 (C), BMI-1 (D) in case of PAE treated samples. Here, GAPDH was used as an internal loading control. All the density analysis is normalized with respect to GAPDH . Here, * $p<0.05$, ** $p<0.01$, *** $p<0.001$; treated with extract vs. control.

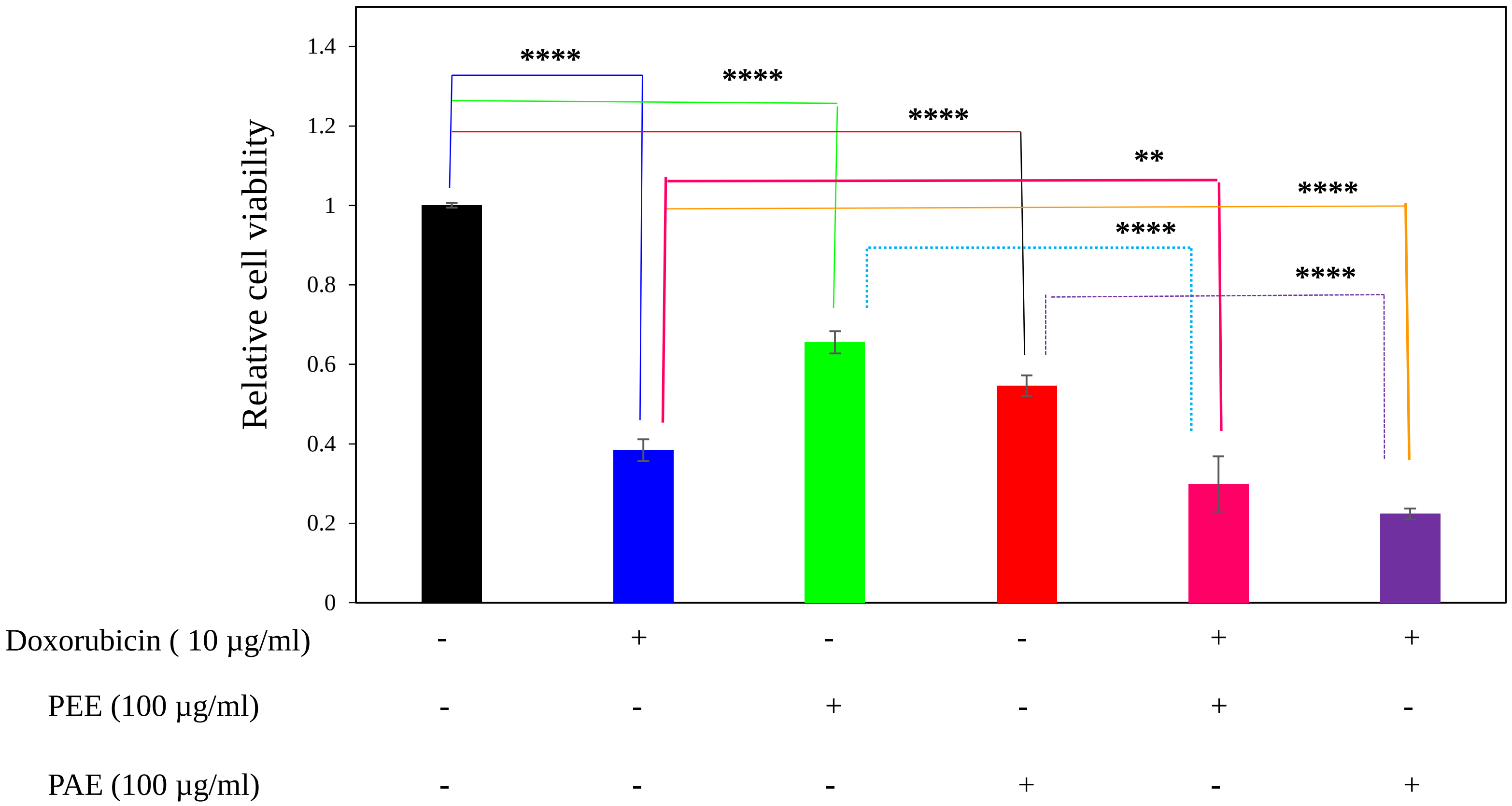

Supplementary Figure S4: Synergistic effect of doxorubicin and plant extracts. Cell viability was measured by MTT assay after 24 hr of treatment of breast cancer MDA-MB-231 cell line with pulp ethanol (PEE) and pulp acetone extract (PAE) with a optimum concentration of 100 µg/ml. Values represent mean ± S.E of triplicate measurements, **p<0.01, and ****p<0.0001, vs. control and treatment.

A B

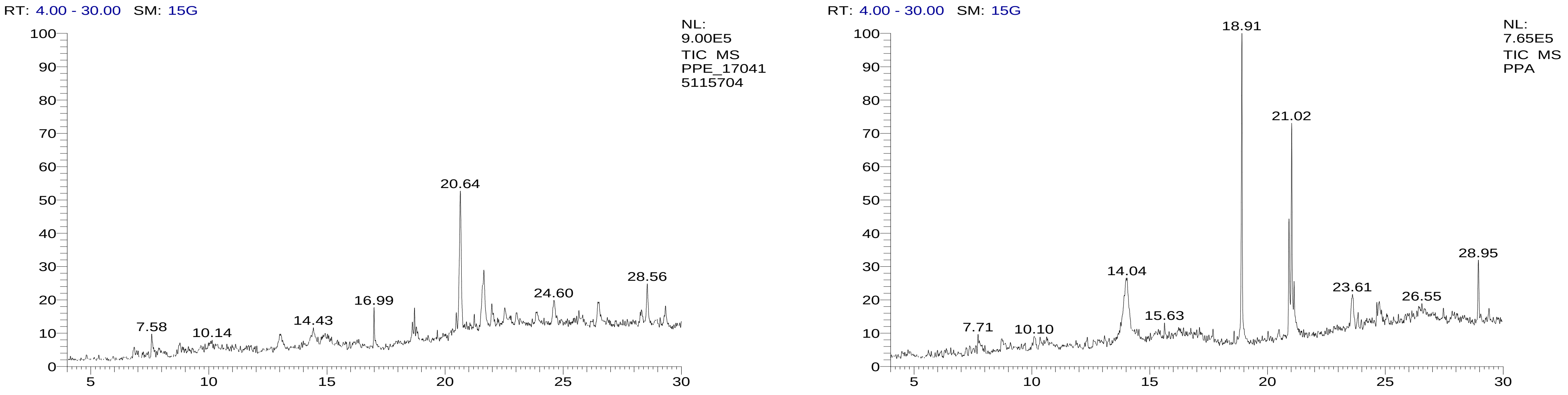

Supplementary Figure S5: GC-MS/MS Plot of the compounds isolated from pulp extract of the plant *Citrullus colocynthis*. Mass spectra analysis of ethanol extract (**A)** and acetone pulp extract (**B**). Peaks represent the compounds on the basis of the relative abundance in the extracts at a given Retention time.
